# Nutritional Risk Indicators for Predicting a Change in Quadriceps Muscle Thickness in Acute Patients with Stroke

**DOI:** 10.31662/jmaj.2021-0107

**Published:** 2021-12-03

**Authors:** Yoji Kokura, Shinta Nishioka

**Affiliations:** 1 Department of Clinical Nutrition, Keiju Medical Center, Nanao, Japan; 2 Department of Medical Nutrition, Graduate School of Human Life Science, Osaka City University, Osaka, Japan; 3 Department of Clinical Nutrition and Food Services, Nagasaki Rehabilitation Hospital, Nagasaki, Japan

**Keywords:** Cerebrovascular Diseases, Malnutrition, Undernutrition, Ultrasound, Controlling Nutritional Status, Geriatric Nutritional Risk Index, Mini Nutritional Assessment - Short Form

## Abstract

**Introduction:**

To date, no studies have assessed the prognostic ability of nutritional indicators to predict changes in quadriceps muscle thickness (QMT). Hence, this study aimed to identify the optimal nutritional indicators for predicting the change in QMT during the acute phase in patients with stroke.

**Methods:**

This retrospective cohort study was a post-hoc analysis of a prospective study in a single hospital. The Geriatric Nutritional Risk Index (GNRI), Controlling Nutritional Status (CONUT), and Mini Nutritional Assessment - Short Form (MNA-SF) were assessed. The primary outcome was the 2-week change in QMT from the time of admission in the paralytic and non-paralytic sides. QMT was evaluated at the rectus femoris and the vastus intermedius in both lower limbs using B-mode ultrasound imaging. The sum of both measurements was defined as QMT. Univariate and multivariate analyses were performed to confirm the effects of nutritional risks assessed by each nutritional indicator on QMT change.

**Results:**

We analyzed 118 patients (mean age, 80.2 ± 8.8 years). No significant difference was found in QMT change in the non-paralytic limbs between the groups stratified based on GNRI and CONUT. However, the difference was significant between the malnourished and normal nutritional status in patients categorized by MNA-SF. After adjusting for potential confounders, a significant association was found between MNA-SF and change in QMT (malnourished vs. normal nutritional status; B = −0.143; 95% confidence interval [CI], −0.254 to −0.031) in the non-paralytic limbs. MNA-SF was not independently associated with change in QMT in the paralytic limb. Furthermore, GNRI and CONUT were not independently associated with change in QMT in both paralytic and non-paralytic limbs.

**Conclusions:**

Although MNA-SF might be useful for predicting the QMT change in non-paralytic limbs, GNRI and CONUT cannot predict the QMT change in either the paralytic or non-paralytic limb.

## Introduction

Older patients with stroke are likely to develop sarcopenia due to ageing and stroke-specific changes in muscle tissues. Sarcopenia is a muscle disease (muscle failure) rooted in adverse muscle changes that accrue over a lifetime ^(1)^. In addition, hemiparalytic stroke leads to various muscle abnormalities. A combination of denervation, disuse, inflammation, remodeling, and spasticity accounts for a complex pattern of muscle tissue phenotypic change and atrophy ^(2)^. Such stroke-specific changes in muscle tissue have been recently categorized as stroke-related sarcopenia ^(3)^. In fact, the muscle size of the lower leg in the paretic side can decrease by 5%-13%. Moreover, the limbs exhibit deficits in muscle size and strength when compared with age-matched non-stroke counterparts ^(4)^. Older people who have suffered from stroke have severe sarcopenia ^(5)^. Therefore, it is important to evaluate sarcopenia and muscle mass in older patients with stroke.

Among the several indicators of skeletal muscles, quadriceps muscle thickness (QMT) is closely associated with post-stroke physical activity ^(6), (7), (8), (9), (10)^. The quadriceps provides stability during the stance phase in the gait cycle and supports normal posture alignment of the knee joint, which is important for gaining gait independence ^(6)^. On the other hand, a decrease in the QMT measured by ultrasound is associated with a decline in physical function in subacute or chronic stroke patients or in older adults ^(7), (8), (9), (10)^. However, the rectus femoris and the vastus intermedius can decrease as early as 1-2 weeks from stroke onset ^(11), (12)^. Because malnutrition may contribute to QMT atrophy in the post-stroke stage ^(13)^, assessing this risk is important to prevent additional loss of muscle mass.

To date, no studies have assessed the predictive capacity of QMT during the acute phase of stroke. The Geriatric Nutritional Risk Index (GNRI) is an objective nutritional risk indicator that uses body weight and serum albumin ^(14)^. Similarly, the Controlling Nutritional Status (CONUT) is a measure of nutritional risk that utilizes serum albumin, total cholesterol, and total lymphocyte count ^(15)^. In patients with stroke, both GNRI and CONUT are independently associated with mortality ^(16), (17)^ and functional prognosis ^(18), (19), (20), (21)^. In addition, the Mini Nutritional Assessment - Short Form (MNA-SF) has been validated in older people ^(22)^, and its improvement has been linked to functional prognosis in patients with stroke ^(23)^. Although GNRI, CONUT, and MNA-SF potentially predict the prognosis of patients with stroke, no longitudinal studies have investigated their ability to forecast the decrease in QMT during the acute phase. Therefore, this study aimed to identify optimal nutritional indicators for predicting the change in QMT during the acute phase in patients with stroke.

## Materials and Methods

### Study design and subjects

This retrospective cohort study was a post-hoc analysis of a prospective study from a single hospital. The details of the original study have been described elsewhere ^(12)^. Patients with stroke who were consecutively admitted to the Keiju Medical Center (a 426-bed core hospital in rural Nanao, Ishikawa Prefecture) in Japan were recruited between December 2016 and November 2018. The inclusion criteria were age ≥65 years and an initial stroke event, which was defined as no history of hospitalization due to stroke. The attending physician determined stroke using computerized tomography or magnetic resonance imaging studies. The exclusion criteria included no paralysis, quadriplegia, a medical history of intractable neurological diseases, a prestroke modified Rankin scale (mRS) score of 5, aggravated clinical condition due to other diseases, death or discharge during follow-up, and involuntary movements that made it difficult to measure QMT via ultrasound. All study participants received nutritional management by a registered dietician based on the 2015 Japanese Guideline for the Management of Stroke ^(24)^. This guideline recommends nutritional assessment in all patients with stroke, and adequate energy and protein should be provided to malnourished patients, those at risk of malnutrition, or those at risk of pressure ulcer. Moreover, enteral nutrition should be provided at an early stage in patients whose oral intake is insufficient for more than 7 days after stroke onset. Individual rehabilitation was facilitated on a daily basis by physical, occupational, and speech-language-hearing therapists. Physical therapists addressed the range of motion of the joint and lower extremity muscle strength and provided standing and walking exercises to improve the functional status. The follow-up period extended for 2 weeks from the time of admission for observing changes in QMT, whereas the follow-up period for observing gait independence degree at discharge was from admission to discharge.

### Measurements

The following basic information was recorded upon admission: age, sex, and premorbid activities of daily living (ADL) using the mRS ^(25)^; comorbidities using the Charlson Comorbidity Index (CCI) ^(26)^; stroke type, location of stroke, and severity of paralysis using the Brunnstrom stroke recovery stage ^(27)^; National Institutes of Health Stroke Scale score ^(28)^; body mass index (BMI); serum concentration of C-reactive protein; albumin; total cholesterol; and total lymphocytes. During the 2-week follow-up, energy and protein intake, as well as days until rehabilitation initiation and days until the estimated duration of rehabilitation, was recorded. To investigate energy and protein intake, the visual estimation method ^(12), (29)^, a method commonly employed in hospitals and other care facilities to evaluate food intake by examining plate waste, was used. Measured meal intake, which was determined by the nurses, was documented on the dietary intake record. Furthermore, energy and protein intake was calculated by registered dieticians. The energy and protein intake from enteral and parenteral nutrition was also evaluated by registered dieticians. Moreover, during follow-up from admission to discharge, gait independence degree at discharge and length of rehabilitation were recorded.

### Nutritional risk indicators and malnutrition screening tool

Nutritional risk was evaluated using three indicators: GNRI ^(14)^, CONUT ^(15)^, and MNA-SF ^(22)^. Blood samples were collected within the first 24 hours of admission. GNRI and CONUT were computed by registered dieticians, who also performed MNA-SF. For patients with consciousness disorders, dysarthria, and/or cognitive impairment, relevant questions with reference to a previous research were posed to the caregiver ^(30)^.

GNRI is an objective malnutrition risk indicator that uses serum albumin, actual body weight, and ideal body weight ^(14)^ and is calculated using the following equation:

GNRI = [1.489 × albumin concentration (g/L)] + [41.7 × (actual body weight/ideal body weight)].

If the actual body weight/ideal body weight was ≥1.0, the ratio was set to 1. The ideal body weight was defined as a BMI of 22.0 kg/m^2^ ^(31)^. Although the original equation of GNRI used the Lorentz formula to evaluate the ideal body weight, the GNRI using a BMI of 22.0 kg/m^2^ demonstrated good agreement with the Lorentz formula ^(31)^. The patients were divided into high nutritional risk (<92) and low nutritional risk (≥92) groups based on GNRI on admission ^(18), (32)^.

The CONUT score is an index of undernutrition calculated from the serum albumin concentration, total peripheral lymphocyte count, and total cholesterol concentration by summing the following scores ^(15)^: (a) albumin concentrations of ≥3.5, 3.0-3.49, 2.5-2.99, and < 2.5 g/dL were scored as 0, 2, 4, and 6 points, respectively; (b) total lymphocyte counts of ≥1600, 1200-1599, 800-1199, and < 800/mm^3^ were scored as 0, 1, 2, and 3 points, respectively; and (c) total cholesterol concentrations of ≥180, 140-179, 100-139, and <100 mg/dL were scored as 0, 1, 2, and 3 points, respectively. The patients were divided into high nutritional risk (5-12) and low nutritional risk (0-4) groups based on the CONUT score on admission ^(17), (21)^.

MNA-SF includes the following six domains: appetite loss (0-2 points), weight loss (0-3 points), mobility (0-2 points), stress/acute disease (0 or 2 points), neuropsychological impairment (0-2 points), and BMI (0-3 points). If the patient's body weight cannot be measured, the calf circumference can be used as a substitute for BMI (0 or 3 points) ^(22)^. According to the total score of MNA-SF, patients were classified into three categories: malnourished (0-7 points), at risk for malnutrition (8-11 points), or normal nutritional status (12-14 points) ^(22)^.

### Outcome measures

The primary outcome was QMT changes in both the paralytic and non-paralytic sides on admission and at the end of follow-up. QMT at the rectus femoris and the vastus intermedius in both lower limbs was assessed using B-mode ultrasound imaging (SonoSite M-Turbo; Fuji film, Tokyo, Japan) with an 8-MHz transducer. The sum of the measurements for the rectus femoris and the vastus intermedius was defined as QMT. All measurements were obtained while the patients were in the supine position, with both knee joints extended fully and relaxed as much as possible and with the transducer positioned midway between the anterior superior iliac spine and the proximal end of the patella ^(11), (12)^. To accurately measure muscle thickness without non-contractile tissue, the measurements were made between the inside edges of the fascia. Ultrasound is a reliable and valid method for assessing muscle size in older adults ^(33)^ and provides a reliable measure of muscle thickness in acute patients with stroke ^(34)^. All measurements were conducted by a single examiner who was well trained in ultrasound measurements. The intraclass correlation coefficient (1, 1) for the test-retest reliability of QMT was 0.952 (95% CI: 0.871-0.987). A physical or occupational therapist evaluated the presence or absence of lower-limb paralysis based on Brunnstrom stage at the start of rehabilitation.

The secondary outcome was gait independence at discharge, which was assessed using the gait score of the Functional Independence Measure tool (FIM) ^(35)^. FIM is one of the most commonly used tools for assessing ADL and includes 13 lower-order items on motor function and 5 lower-order items on cognitive function ^(35)^. FIM gait scores range from 1 (total assistance) to 7 (complete independence). The level of physical assistance required for walking is represented by an FIM gait score of 1-5 and that for independent walking by a score of 6 or 7 ^(35)^. In this study, gait independence was defined as an FIM gait score of 6 or 7. To ensure accuracy, lower-limb paralysis measurements at the start of rehabilitation and the walk FIM scores at discharge were determined by a physical therapist or occupational therapist. Brunnstrom stage ≤ 6 was considered to indicate paresis.

### Statistical analysis

JMP 11.2.1 software (SAS Japan, Tokyo, Japan) was applied for statistical analyses. Continuous and ordinal data were presented as mean ± standard deviation and median [25^th^, 75^th^ percentiles], respectively. Categorical data were expressed as the absolute value and percentage. Patients were stratified into two or three groups based on the nutritional risk indicators as follows: high nutritional risk (<92) and low nutritional risk (≥92) based on GNRI; high nutritional risk (5-12) and low nutritional risk (0-4) based on CONUT; and malnourished (0-7), at risk of malnutrition (8-11), and normal nutritional status (12-14) based on MNA-SF. Furthermore, patients were stratified into two groups based on gait independence at discharge. Mann-Whitney U-test and Kruskal-Wallis test were used to compare the QMT at admission and after 2 weeks as well as the changes in different nutritional categories because these variables were non-normally distributed. The Mann-Whitney U-test, chi-square test, and Fisher's exact test were used to analyze the differences in gait independence or gait non-independence. Multiple linear regression analyses were performed to confirm the effects of nutritional risks as assessed by each nutritional indicator on QMT change over 2 weeks. For each nutritional indicator, three multiple regression models were developed, with change in QMT as the dependent variable. We assumed that the following variables were potential confounders as all of these are theoretically related to functional outcomes ^(1), (11), (12), (36)^: age, sex, premorbid mRS, CCI, National Institutes of Health Stroke Scale, energy intake during the 2-week period, estimated duration of rehabilitation, and QMT at admission. These variables were forcibly included in the models as independent variables. Change in QMT, age, premorbid mRS, CCI, National Institutes of Health Stroke Scale, the 2-week energy intake, estimated duration of rehabilitation, and QMT at admission were the continuous variables, and sex was the categorical variable. The absence of multicollinearity among all variables was confirmed if the variance inflation factor was <2. Multivariate logistic regression analysis was performed with gait independence at discharge as the objective variable and the 2-week change in QMT and each covariate as the explanatory variables. Potential confounders were age, stroke type, NIHSS, and length of rehabilitation ^(37), (38), (39)^. P < 0.05 was considered statistically significant.

### Ethical consideration

In the previous study from which the data for the current study were derived, informed consent was obtained from all patients or their legal guardians ^(12)^. The current study was conducted in accordance with the Declaration of Helsinki and was approved by the Keiju Medical Center Ethics Committee (Ref No. 2020-4-1). Because of the anonymous nature of the data, the requirement for informed consent was waived. Instead, we provided an opt-out option to allow the patients to withdraw from the database. The patients could withdraw from the study at any time if they wished to do so.

## Results

Overall, 157 patients fulfilled the inclusion criteria. Among them, the following were excluded: one due to a medical history of intractable neurological diseases, six due to non-paralysis, nine due to quadriplegia, three due to the score on the mRS, three due to the inability of having their QMT measured because of involuntary movements, three due to death during the 2-week follow-up, one due to the aggravation of the clinical condition because of other diseases, and thirteen due to discharge before completing the follow-up. Finally, 118 patients were analyzed in this study (mean age, 80.2 ± 8.8 years; 61 men [51.7%]). Median length of days from stroke onset to hospital admission was 0 (interquartile range [IQR]: 0-0) days.

Table 1 shows the characteristics of all patients. Ninety-three (78.8%) patients were admitted for cerebral infarction, and twenty-five (21.2%) had intracerebral hemorrhage. The ages of 65 and 98 years were the minimum and maximum, respectively, for the patients included in the study.

**Table 1.** Patient Characteristics.

Variable	Overall (n = 118)
Age (year)	80.2 (8.8)
Male sex, n (%)	61 (51.7)
Prestroke modified Rankin scale	0 [0, 1]
Charlson Comorbidity Index	1 [0, 1]
Stroke type, n (%)	
Cerebral infarction	93 (78.8)
Intracerebral hemorrhage	25 (21.2)
Location of stroke, n (%)	
Supratentorial	110 (93.2)
Infratentorial	8 (6.8)
Paralysis, n (%)	
Right hemiplegia	59 (50.0)
Left hemiplegia	59 (50.0)
Brunnstrom stage of paralytic side	5 [3, 6]
National Institutes of Health Stroke Scale	5 [3, 10]
C-reactive protein, mg/dL	0.16 [0.06, 0.34]
Serum albumin, g/dL	3.9 [3.7, 4.2]
Total cholesterol, mg/dL	191 (38)
Total lymphocytes, /mm3	1434 [1001, 1868]
BMI, kg/m^2^	23.1 (3.6)
Energy intake during 2 weeks,	
kcal/actual body weight/day	22.4 (7.6)
Oral intake	19.8 (10.0)
Enteral nutrition	1.2 (4.0)
Parenteral nutrition	1.3 (2.4)
Protein intake during 2 weeks,	
g/actual body weight/day	0.9 (0.3)
Oral intake	0.8 (0.4)
Enteral nutrition	0.1 (0.2)
Parenteral nutrition	0.1 (0.1)
Days from admission to the initiation of rehabilitation	1 [1, 2]
Estimated time of rehabilitation dose during 2 weeks, minute/day	107 [85, 122]

Data are expressed as mean (standard deviation), median [interquartile range], or n (%). BMI, body mass index; CONUT, Controlling Nutritional Status; GNRI, Geriatric Nutritional Risk Index; MNA-SF, Mini Nutritional Assessment - Short Form

Table 2 shows comparisons of QMT between the groups based on nutritional status or risk. When the patients were categorized by GNRI or CONUT, those at high nutritional risk had significantly lower QMT in both paralytic and non-paralytic limbs at admission and after 2 weeks than those at low risk (P < 0.05). The malnourished patients categorized by MNA-SF had significantly lower QMT at admission and after 2 weeks than those at risk of malnutrition and with normal nutritional status (P < 0.001). In the paralytic limb, there was no significant difference in QMT change among different nutritional risk groups based on GNRI, CONUT, or MNA-SF. In the non-paralytic limbs, there was no significant difference in the QMT change among the groups stratified by GNRI and CONUT, but there was a significant difference between malnourished and normal nutritional status when patients were categorized based on MNA-SF.

**Table 2.** Patients’ Nutritional Risk as Assessed Using the Three Nutritional Indicators.

Limb	Nutritional risk	n (%)	QMT at admission (cm)		QMT after 2 weeks (cm)		Change in QMT (cm)	
			Median (IQR)	P value	Median (IQR)	P value	Median (IQR)	P value
Paralytic	GNRI							
	High risk (<92)	20 (17.0)	1.80 [1.16, 2.33]	<0.001^1^	1.44 [1.31, 2.00]	<0.001^1^	−0.17 [−0.46, 0.03]	0.561^1^
	Low risk (≥92)	98 (83.0)	2.74 [2.27, 3.23]		2.49 [2.04, 2.98]		−0.24 [−0.50, 0.02]	
	CONUT							
	High risk (5-12)	11 (9.3)	1.85 [1.67, 2.34]	0.017^1^	1.88 [1.45, 2.15]	0.004^1^	−0.28 [−0.46, 0.08]	0.853^1^
	Low risk (0-4)	107 (90.4)	2.71 [2.22, 3.21]		2.42 [1.95, 2.90]		−0.42 [1.95, 2.90]	
	MNA-SF							
	Malnourished (0-7)	48 (40.7)	2.30 [1.83, 2.86]*^3^,**^4^	<0.001 ^2^	2.03 [1.46, 2.42]**^3^,**^4^	<0.001^2^	−0.33 [−0.58, 0.04]	0.181^2^
	At risk of malnutrition (8-11)	60 (50.8)	2.75 [2.29, 3.18]		2.59 [2.00, 3.01]		−0.18 [−0.44, 0.09]	
	Normal nutritional status (12-14)	10 (8.5)	3.32 [2.69, 3.72]		3.16 [2.51, 3.48]		−0.16 [2.51, 3.48]	
Non-paralytic	GNRI						
	High risk	―	1.89 [1.37, 2.52]	<0.001^1^	1.77 [1.39, 2.01]	<0.001^1^	−0.12 [−0.33, 0.08]	0.624^1^
	Low risk	―	2.74 [2.37, 3.27]		2.61 [2.13, 3.14]		−0.17 [−0.39, 0.03]	
	CONUT							
	High risk	―	2.08 [1.52, 2.80]	0.014^1^	1.78 [1.60, 2.01]	0.004^ 1^	−0.04 [−0.56, 0.08]	0.835^1^
	Low risk	―	2.65 [2.24, 3.27]		2.52 [2.03, 3.02]		−0.17 [−0.38, 0.03]	
	MNA-SF							
	Malnourished At risk of malnutrition	―	2.42 [1.79, 3.00]**^4^	0.003 ^2^	1.97 [1.60, 2.75] **^3^,**^4^	<0.001 ^2^	−0.25 [−0.45, −0.08]*^3^	0.007^2^
		―	2.73 [2.39, 3.22]		2.61 [2.22, 3.22]		−0.08 [−0.36, 0.12]	
	Normal nutritional status	―	3.45 [2.64, 3.76]		3.20 [2.52, 3.90]		−0.03 [−0.32, 0.16]	

^1^ Mann-Whitney U-test; ^2^ Kruskal-Wallis test; ^3^ Significant differences compared with at risk of malnutrition by Steel-Dwass test; *P < 0.05, **P < 0.01; ^4^ Significant differences compared with normal nutritional status by Steel-Dwass test; *P < 0.05, **P < 0.01. CONUT, Controlling Nutritional Status; QMT, quadriceps muscle thickness; GNRI, Geriatric Nutritional Risk Index; IQR, interquartile range; MNA-SF, Mini Nutritional Assessment - Short Form

In addition, of 118 patients, 11 who died until discharge were excluded. Therefore, data pertaining to 107 patients (180 men and 160 women; mean age, 76.6 ± 12.4 years) were extracted for the analysis of gait independence degree at discharge. Table 3 shows the univariate analysis of gait independence degree at discharge. A significant difference was found in the 2-week change in QMT in the non-paralytic limb between the independent and non-independent groups (P < 0.001).

**Table 3.** Univariate Analysis of Gait Independence Degree at Discharge.

	Gait independence degree		
	Independent	Non-independent	P value
	n = 61	n = 46	
Age (year)	76 [71, 83]	85 [78, 91]	<0.001^1^
Stroke type, n (%)			
Cerebral infarction	51 (83.6)	10 (16.4)	0.085^2^
Intracerebral hemorrhage	32 (69.6)	14 (30.4)	
National Institutes of Health Stroke Scale	3 [2, 5]	10 [5, 17]	<0.001^1^
Length of rehabilitation, days	41 [19, 66]	91 [49, 127]	<0.001^1^
Length of hospitalization	42 [20, 69]	93 [52, 129]	<0.001^1^
Gait independence at the initiation of rehabilitation, (%)	12 (19.7)	0 (0)	0.001^3^
Change in QMT during 2 weeks			
Paralytic	−0.18 [−0.45, −0.08]	−0.33 [−0.63, −0.01]	0.105^1^
Non-paralytic	−0.04 [−0.30, 0.10]	−0.31 [−0.56, −0.11]	<0.001^1^

^1^ Mann-Whitney U-test; ^2^ χ^2^-test; ^3^ Fisher’s exact test. Data are expressed as median [interquartile range] or n (%). QMT, quadriceps muscle thickness

Table 4 presents the results of the multiple regression analyses. After adjusting for potential confounders, a significant association between MNA-SF and QMT change (malnourished vs. normal nutritional status; B = −0.143; 95% CI, −0.254 to −0.031) was noted in the non-paralytic limb. In contrast, MNA-SF was not independently associated with change in QMT in the paralytic limb. Similarly, GNRI and CONUT were not independently associated with change in QMT of the paralytic and non-paralytic limbs.

**Table 4.** Multivariate Analysis of QMT Change during the 2-week Follow-up and Nutritional Risk at Admission Using Three Nutritional Indicators.

Limb	Nutritional risk	B	P value	95% CI		β
				Lower	Upper	
Paralytic	GNRI					
	Low risk	Reference				
	High risk	−0.106	0.070	−0.221	0.009	−0.187
	CONUT					
	Low risk	Reference				
	High risk	−0.049	0.464	−0.180	0.082	−0.068
	MNA-SF					
	Normal nutritional status	Reference				
	At risk of malnutrition	0.047	0.386	−0.060	0.153	0.072
	Malnourished	−0.108	0.109	−0.241	0.024	−0.162
Non-paralytic	GNRI					
	Low risk	Reference				
	High risk	−0.013	0.803	−0.113	0.088	−0.027
	CONUT					
	Low risk	Reference				
	High risk	−0.022	0.697	−0.137	0.092	−0.038
	MNA-SF					
	Normal nutritional status	Reference				
	At risk of malnutrition	0.037	0.416	−0.053	0.128	0.070
	Malnourished	−0.143	0.013*	−0.254	−0.031	−0.258

B, partial regression coefficient; β, standardized partial regression coefficient; CI, confidence interval; CONUT, Controlling Nutritional Status; QMT, quadriceps muscle thickness; GNRI, Geriatric Nutritional Risk Index; MNA-SF, Mini Nutritional Assessment - Short Form. *P < 0.01 Multiple linear regression analyses were performed with the QMT change during 2 weeks as the dependent value and the nutritional indicator as the independent value. All models were adjusted for age, sex, premorbid modified Rankin scale, Charlson Comorbidity Index, National Institutes of Health Stroke Scale, energy intake during two weeks, estimated time of rehabilitation dose, and QMT at admission. Adjusted R^2^ for each model: 0.305 in the GNRI, 0.292 in the CONUT, and 0.297 in the MNA-SF of the paralytic limb; 0.202 in the GNRI, 0.204 in the CONUT, and 0.260 in the MNA-SF of the non-paralytic limb (all P < 0.01)

Multivariate logistic regression analysis revealed that the 2-week change in QMT in the non-paralytic limb was associated with gait independence at discharge (OR = 12.710; 95% CI, 1.722-120.520; P = 0.017) (Table 5).

**Table 5.** Multivariate Analysis of Gait Independence Degree at Discharge.

	Model 1		Model 2	
	OR (95% CI)	P value	OR (95% CI)	P value
Age (year)	0.908 (0.842-0.971)	0.007	0.905 (0.835-0.970)	0.008
Stroke type, cerebral infarction	0.976 (0.218-4.041)	0.973	0.532 (0.144-1.817)	0.982
National Institutes of Health Stroke Scale on admission	0.819 (0.708-0.920)	.003	0.822 (0.713-0.920)	0.002
Length of rehabilitation, days	0.975 (0.957-0.990)	0.003	0.978 (0.959-0.993)	0.016
Change in QMT during 2 weeks (paralytic limb)	0.98 (0.212-4.611)	0.981		
Change in QMT during 2 weeks (non-paralytic limb)		12.710 (1.722-120.520)	0.017

OR, odds ratio; CI, confidence interval; QMT, quadriceps muscle thickness Model 1: Adjusted for age, stroke type, cerebral infarction, National Institutes of Health Stroke Scale on admission, length of rehabilitation days, and change in QMT during two weeks; Model 2: Adjusted for age, stroke type, cerebral infarction, National Institutes of Health Stroke Scale on admission, length of rehabilitation days, and change in QMT during two weeks (non-paralytic limb)

## Discussion

This study aimed to determine the optimal nutritional indicators for predicting the change in QMT in patients with acute stroke. The study revealed two important conclusions regarding evidence for change in QMT. First, MNA-SF may be useful for predicting the QMT change in non-paralytic limbs. Second, GNRI and CONUT cannot predict the change in QMT in either the paralytic or non-paralytic limb.

MNA-SF may be beneficial in predicting QMT change in the non-paralytic limb. In previous studies, the MNA-SF score appropriately predicted low lean muscle mass index (area under the curve = 0.762; P < 0.001) ^(40)^. In patients with heart failure, the MNA-SF score is also an independent variable for appendicular skeletal muscle mass index ^(41)^. In addition, the MNA score is linearly related to muscle mass in non-institutionalized older people (r = 0.72; P < 0.001) ^(42)^. The MNA-SF subscores indicate not only nutritional intake but also decreased mobility and cognitive function. Poorer cognitive function in people with type 2 diabetes, including after stroke, was associated with decreased lower-extremity skeletal muscle mass ^(43)^. In addition, poorer lower mobility was linked to a smaller mid-thigh muscle area in older men and women ^(44)^. Muscle atrophy is thus more likely to occur because of lower physical function. Therefore, MNA-SF can predict the change in muscle mass caused by insufficient food intake and immobilization. Moreover, MNA-SF can evaluate nutritional risk without the use of biomarkers, and it may be useful for predicting QMT change in the non-paralytic limb of patients with stroke. In contrast, MNA-SF did not predict the QMT change in the paralytic limbs, which might be because muscle atrophy was due to a non-nutritional cause after stroke. Stroke-related sarcopenia is a multifactorial syndrome that is characterized by impaired neurovegetative control, loss of motoneurons and degeneration of neuromuscular junctions, systemic catabolic-anabolic imbalance, and local muscle metabolic alterations ^(3)^. Because MNA-SF does not assess all aspects of stroke-related sarcopenia, it would be unsuitable for predicting QMT changes in non-paralyzed limbs after stroke.

Another important finding of this study is that GNRI and CONUT did not predict the change in QMT. Previous studies have reported that lower GNRI was significantly associated with low lean mass ^(45)^, low muscle mass ^(46)^, and low appendicular skeletal muscle ^(46)^. However, our literature search failed to reveal any relationship between GNRI and change in skeletal muscle mass. The biomarkers constituting both GNRI and CONUT are not suitable for predicting muscle mass loss as they can fluctuate because of inflammation or fluid status, regardless of the nutritional status. In particular, serum albumin level is affected by various factors such as redistribution of ovalbumin in the interstitium, plasma volume expansion, infection, burns, fluid overload, hepatic failure, cancer, and nephrotic syndrome ^(47), (48)^. In fact, serum albumin, total lymphocyte count, and total cholesterol, which are components of GNRI and CONUT, were weakly correlated with skeletal muscle index in patients with gastric cancer. However, there was no significant correlation between skeletal muscle index and CONUT scores ^(49)^. In patients with stroke, GNRI and CONUT were independent predictive factors of all-cause mortality ^(16), (17)^ and functional prognosis ^(18), (19), (20), (21)^. Although both tools may be helpful in predicting mortality, they are insufficient for predicting the change in muscle mass.

The 2-week change in QMT in non-paralytic limbs was associated with gait independence at discharge. Muscle strength in the lower limbs was linked to the reacquisition of walking ability ^(37)^. In stroke patients, QMT represents muscle strength ^(50)^. In addition, lower rectus femoris thickness is related to a slower walking speed and shorter walking distance in older adults ^(51)^. Thus, a decrease in QMT indicates decreased muscle strength in lower limbs and, thus, delayed recovery of gait ability.

This study has several limitations. First, the study subjects were limited to those with an initial stroke event. Therefore, whether the results apply to patients with recurrent stroke remains unclear. Second, because this study was a post-hoc analysis of a previous study, the sample size was limited. Third, our study was conducted at a single center. A multicenter prospective study is needed to generalize our results to a wider population.

One of the strengths of our study is that ours is the first study to observe changes in muscle mass after stroke using ultrasound and to evaluate the predictiveness of change in combination with nutritional indicators. The results may contribute toward the nutritional care of patients with stroke as nutritional indicators concurrently predict the decrease in muscle mass. Future research to prevent the development of sarcopenia is warranted.

MNA-SF may be useful for predicting QMT change in the non-paralytic limb. In contrast, GNRI and CONUT cannot predict the change in QMT in either the paralytic or non-paralytic limb. In older patients with stroke, using a reliable and valid malnutrition screening tool to predict QMT changes during the acute phase may be beneficial.

## Article Information

### Conflicts of Interest

None

### Sources of Funding

This work was supported by a research Grant-in-Aid for Scientific Research C (no. 16K12059) from the Japan Society for the Promotion of Science.

### Acknowledgement

We are grateful to Mayumi Kato, and the staff of the Keiju Medical Centre for their advice and expertise regarding this study. This work was supported by a research Grant-in-Aid for Scientific Research C (no. 16K12059) from the Japan Society for the Promotion of Science.

### Author Contributions

Yoji Kokura was responsible for study conception and design, data collection and interpretation, and drafting and revision of the manuscript. Shinta Nishioka was responsible for study conception and design, data interpretation, and revision of the manuscript. Yoji Kokura and Shinta Nishioka contributed equally to this work. All authors approved the ﬁnal version of the manuscript.

### Approval by Institutional Review Board (IRB)

This retrospective cohort study was approved by the Ethics Committee of the Keiju Medical Center (approval number: 2020-4-1).
